# Bridging the Blood-Brain Barrier: New Methods Improve the Odds of Getting Drugs to the Brain Cells That Need Them

**DOI:** 10.1371/journal.pbio.0050169

**Published:** 2007-06-12

**Authors:** Dan Ferber

## Abstract

Researchers developing drugs to treat brain disorders face a major hurdle: crossing the blood-brain barrier. What will it take to translate billions of dollars of research into therapeutic success?

When it comes to brain disease, William Pardridge knows the numbers, and he'll rattle them off at the drop of a hat. One in five: the number of people with a disorder of the brain or central nervous system. Half a trillion dollars: what we'll spend in the year 2020 to treat Alzheimer disease and stroke alone, and about what we now spend annually on national defense. And then there's the number of good drugs available, after decades of research, to help brain cells survive in patients with those disorders: zero.

Brain diseases by the dozens lack effective therapies, but it's not for lack of trying, says Pardridge, a professor of medicine at the University of California, Los Angeles (United States): Pharmaceutical companies have failed to deliver effective new brain drugs, despite huge potential markets for Alzheimer disease, stroke, Parkinson disease, brain infections, and more. What's more, “NIH is funding billions of dollars a year on molecular neuroscience research on the assumption that therapies for these diseases will be translated from the basic biological research,” but that assumption is proving to be false, he says.

**“The walls of capillaries in peripheral tissues are like Swiss cheese, whereas the walls of capillaries in the brain are like cheddar cheese” —William Pardridge**

Those billions fund research that sheds light on the basic biology of brain diseases, which in many cases are still poorly understood. But the real problem in developing brain drugs, Pardridge maintains, is the blood-brain barrier, the super-tight seal in the walls of the brain's capillaries that protects the brain from brain-disrupting blood compounds like amino acids (which act as neurotransmitters), alkaloid toxins from plants, and other toxins.

The same barrier, however, keeps out potentially beneficial drugs. Only 2% of small-molecule compounds enter the brain on their own, even though they cross easily into other tissues. (Molecules are considered small if they're less than 400 daltons, roughly the size of sucrose.) No large molecules cross—no monoclonal antibodies, no gene therapies, no antisense and RNA interference compounds, no recombinant proteins. “We have come up with a number of potential agents that could benefit humans,” says Thomas Jacobs, a program officer at the US National Institute for Neurological Disorders and Stroke (NINDS) who directs both stroke and blood-brain barrier research, “but we run into a major roadblock when we try to deliver them to the nervous system.”

**Figure:**
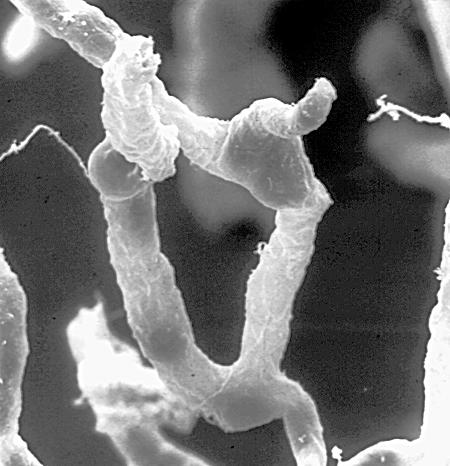
A network of capillaries supply brain cells with nutrients. Tight seals in their walls keep blood toxins—and many beneficial drugs—out of the brain.

And while biology is a major roadblock, the more important problem is that the field of blood-brain barrier drug delivery has been neglected, underfunded, and underdeveloped, says Pardridge. “Not a single large pharmaceutical company in the world today has a blood-brain barrier drug-targeting program,” he says. And “not a single academic neuroscience program in the United States teaches blood-brain barrier drug delivery.”

Unhappy with the progress, a few years ago Pardridge founded Armagen, a startup biotech company devoted to finding better ways to deliver drugs across the blood-brain barrier. Now, through the work of Pardridge's team and a small group of other researchers, the field of blood-brain barrier drug delivery is showing new signs of life.

## Keeping the Brain Clean

The first evidence for a blood-brain barrier arose in the late 1800s, when the German immunologist Paul Ehrlich injected aniline dyes into a rat's bloodstream in an effort to stain tissue. Every tissue in the rats stained crimson—except the brain and spinal cord. Later, his student Edwin Goldmann showed that dye injected into the cerebrospinal fluid stained cells throughout the brain, but did not escape to stain peripheral tissues. Taken together, the two experiments showed that a tight seal existed between blood and brain.

**Figure:**
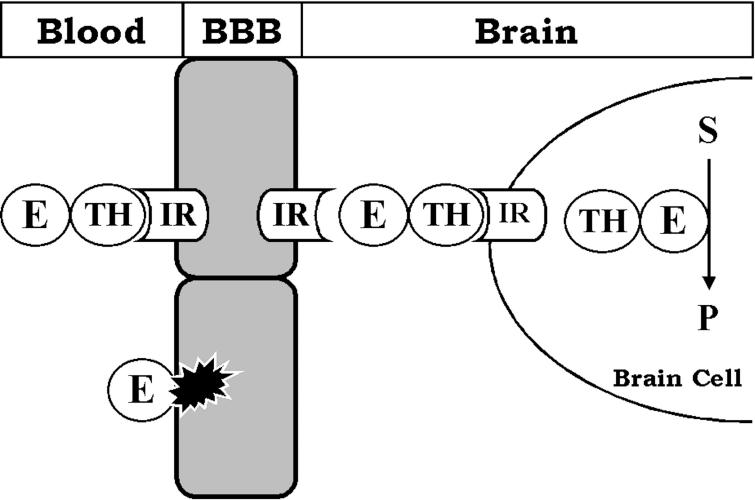
Trojan horse molecules, such as monoclonal antibodies, can carry drugs (E) to brain cells.

The nature of that seal did not become clear until the late 1960s, when electron micrographs of brain capillaries revealed that they differ from capillaries in the rest of the body. The endothelial cells that make up the walls of brain capillaries form tight junctions, preventing the passage of fluid between individual cells. The cells also have fewer fenestrations, or tiny holes in their membranes, and they exhibit much less cellular drinking than endothelial cells in other tissues, reducing leakage. They also have nonselective pumps called P-glycoproteins to remove molecules that manage to sneak in. “The walls of capillaries in peripheral tissues are like Swiss cheese, whereas the walls of capillaries in the brain are like cheddar cheese,” Pardridge says.

That tight seal “ensures the neuronal environment is crystal clean,” Pardridge says, which prevents the neural pandemonium that would ensue if blood compounds could easily enter brain tissue. But the tradeoff comes when drug researchers—and patients—want a drug to heal an ailing brain.

“To get drugs into the brain to treat disease, you're going up against millions of years of development in man to keep out toxins and keep the brain healthy,” says medical pharmacologist Thomas Davis of the University of Arizona College of Medicine (United States). To deliver drugs to treat diseases of the central nervous system, “you try to fake the brain out,” Davis says.

## Strategy Debates

Faking the brain out, however, is easier said than done, and according to Pardridge and other academic researchers, pharmaceutical companies have mostly botched the job. As Pardridge describes it, the pharmaceutical industry employs armies of medicinal chemists to develop hundreds of candidate drug compounds that they predict will cross the blood-brain barrier. Then they screen the promising compounds for activity in test-tube and cell-culture experiments, and then in rodents and other animals. Once inside the brain, such a drug might promote neuron growth, inhibit neuron degeneration, or stimulate repair. But often the companies find out only during clinical trials that a drug fails to cross the human blood-brain barrier.

That whole strategy is backwards, Pardridge maintains. Time after time, companies have learned too late that not enough of the drug enters the brain to benefit patients. “A lot of companies have the attitude that we'll cross the blood-brain barrier problem when we get to it. That's moronic,” he says. Other academic researchers put it a tad more gently, but basically agree. “Pharmaceutical companies wait until after drugs are developed to see if they get into the brain,” Davis says. “It's a huge error.”

Representatives of Eli Lilly and Company, Sanofi-Aventis, Merck, Amgen, and GlaxoSmithKline declined to speak to *PLoS Biology* for this story. But Steve Lederer, a spokesman for Pfizer, says that Pardridge's depiction is a caricature of their process. Clinical trials are so expensive that “it would be insanity” to develop drugs and do clinical trials on them without knowing if they crossed the blood-brain barrier, he says.

In addition to creating small molecules and screening them to see if they nurture or save neurons, Lederer says, Pfizer uses “very advanced computational modeling techniques” to predict, based on a molecule's size and shape, whether it will cross the barrier, Lederer says. They choose only the compounds that computer modeling and animal experiments predict will work in the human brain. Then they do imaging studies on humans with tiny amounts of labeled drug to see whether it enters the brain. Finally, they use sophisticated biomarkers of activity during small phase I trials. In a trial of an experimental drug for Parkinson disease, the company used speech recognition technology to detect subtle changes on patient's vocal cords, a sensitive measure of effectiveness. “We never progress to clinical trial unless we have a good understanding of what a drug is doing,” Lederer says.

**“We have come up with a number of potential agents that could benefit humans, but we run into a major roadblock when we try to deliver them to the nervous system.” —Tom Jacobs**

Pardridge says that NIH and the academic neuroscience community share the blame for the failures of central nervous system drug development for focusing on basic molecular neuroscience research rather than important but less sexy translational research into the blood-brain barrier. NINDS' Jacobs agrees that more could be done: “A Manhattan-type project on the blood-brain barrier would make tremendous strides, but the likelihood of that happening . . . there's some difficulty.”

Nonetheless, the field has recently picked up, with help from the NIH, Jacobs says. The NINDS, the National Institute of Mental Health, and the National Institute of Aging recently completed a three-year blood-brain barrier initiative specifically geared to coming up with new ways of delivering compounds to the brain. In October 2006, those three institutes and six others issued a new request for proposals with the same goal as part of NIH's Neuroscience Blueprint Initiative. In the summer of 2006, academic blood-brain barrier researchers formed the International Brain Barriers Society (IBBS) to encourage “scientific and clinical research on the biological barriers in the central nervous system,” and in March of 2007, the organization hosted 125 leaders in the field at a meeting in Stevenson, Washington, United States, to scope out the research challenges ahead.

## Squeezing Through

Pardridge, for his part, has been scoping out those challenges for nearly four decades. His startup company, Armagen, has developed what Davis calls “probably the best technology in the world” to deliver drugs across the barrier. The technology, which has so far been tested only in animals, makes use of endothelial-cell transport proteins that normally move nutrients, hormones, or other peptides into the brain. Its design resembles the Trojan horse of ancient mythology: A monoclonal antibody looks familiar enough to convince the transporter to move it into brain tissue. The drug hitches a ride on the antibody into the brain tissue, then moves to diseased or damaged neurons to help them heal.

**Figure:**
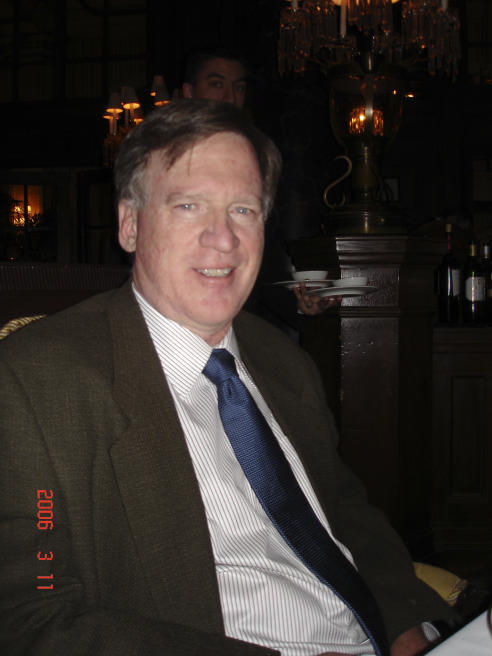
William Pardridge and the company he founded, Armagen, ferry drugs across the blood-brain barrier by attaching them to other molecules that act like a Trojan horse.

Last year, the company's most advanced Trojan-horse drug, called AGT-120, prevented stroke-induced brain damage in rats. (Such a drug is desperately needed: no drug is approved by the US Food and Drug Administration (FDA) to nurture brain cells in the critical hours following a stroke.) AGT-120 is a monoclonal antibody, an antibody specifically produced to bind a single part, or epitope, of a protein. AGT-120 binds a capillary endothelial cell transporter that delivers a protein called transferrin into the brain, and it binds without interfering with the transporter's activity. The payload is a peptide called brain-derived neurotrophic factor (BDNF), one of a large class of peptides called neurotrophins that nourish neurons.

In a 2006 paper in *Brain Research*, Pardridge and research scientist Yun Zhang reported giving a rat-specific version of AGT-120 intravenously to animals that had an artificially induced ischemic stroke. AGT-120 reduced the volume of damaged cerebral tissue by 62% compared with intravenous BDNF alone, and it enabled the rats to balance on a spinning drum more than three times as long as rats treated only with BDNF, thereby proving that the Trojan horse strategy protected the animals from stroke-induced brain damage. The company will apply for FDA approval this year for a phase I clinical trial of AGT-120, and it's developing seven other drugs for currently untreatable brain disorders, such as Alzheimer disease, Parkinson disease, mental retardation, and inborn errors of metabolism.

Trojan horses can also deliver large molecules that would never stand a chance of crossing the barrier otherwise, including enzymes, monoclonal antibody drugs, RNA interference, and gene therapy (via Trojan horse–linked liposomes).

**“A lot of companies have the attitude that we'll cross the blood-brain barrier problem when we get to it. That's moronic.” —William Pardridge**

**“It would be insanity to do that.” —Steve Lederer, spokesman for Pfizer**

Trojan-horse technologies are “very promising,” Jacobs says, but they “may not be a panacea, because they're very specific and very specialized.” What's more, says neuroscientist Maiken Nedergaard of the University of Rochester Medical Center (United States), the blood-brain barrier receptors exist elsewhere in the body, so “you get tremendous uptake in other organs.”

Neurosurgeons have developed alternative methods of disrupting the barrier, some of which have been used clinically. Some neurosurgeons have treated brain tumors by infusing small volumes of fluid directly onto the affected parts of the brain. Early results were promising, but two recent phase III trials using the method, called convection-enhanced delivery, have failed. Edward Neuwelt, a neurosurgeon and neuroscientist at Oregon Health Sciences University and Portland Veteran's Administration Hospital (United States), opens the blood-brain barrier in patients with brain tumors by snaking a catheter directly into the artery that supplies the affected part of the brain, then injecting a sugary solution, which sucks water out of endothelial cells. That shrinks them for up to half an hour and allows compounds as big as an antibody to squeeze through otherwise impervious tight junctions.

In a 2001 study, Neuwelt reported that the osmotic disruption method enabled 42% of 74 patients with primary central nervous system lymphoma to live at least five years longer than they would without treatment. Moreover, the method did not cause dementia, a common side effect of the chemotherapy and radiation that's typically used to save such patients. Neuwelt and colleagues plan to present similar results from a 175-patient, multicenter trial at the American Society of Clinical Oncology meeting in June. The osmotic disruption method has drawbacks, however, admits Neuwelt. Only a few hospitals have neurosurgeons who have the special training needed.

Neuroscientists have developed several other targeting methods that show some promise in animals, Jacobs says. Some researchers deliver drugs via nasal inhalation, which deliver both large- and small-molecule drugs directly to the brain via the olfactory and trigeminal nerves, which relay sensory signals from the nose and mouth. Others have tested localized application of ultrasound to open up small areas of vasculature without causing tissue damage; Jacobs says the method “one day could help.” Neuroscientists have also used polymer nanoparticles to encapsulate and deliver a wide range of drugs, or they have loaded brain-penetrating macrophages with drug-linked nanoparticles.

Still others, including many drug companies, argue that no targeting is really needed, because the blood-brain barrier opens naturally during many diseases. “But to rely on that as a way of getting drugs into the brain is not so rigorous,” Jacobs says. Indeed, despite all the research, “I don't think there exists any way of selectively targeting the brain right now,” says Nedergaard, who cochaired the brain drug delivery section of the March International Brain Barriers Society conference.

That's one reason neuroscientist Damir Janigro of Cleveland Clinic Foundation (United States) has spent years developing more and more realistic in vitro models of the human blood-brain barrier. The latest such model, called dynamic in vitro blood-brain barrier, consists of a porous hollow-fiber tube that mimics a capillary wall. Endothelial cells line the inside wall of the tube; astrocytes, which regulate the blood brain barrier, cover the outside of the tube, and blood flow is mimicked by flowing media that contains white blood cells. The model, which is being produced by a spinoff company to sell to drug companies and researchers, can reproduce the properties of the healthy human blood-brain barrier as well as the altered blood-brain barrier of patients with multidrug-resistant epilepsy, according to a paper published online in February, 2007, in *Epilepsia*. It will be sold to drug companies to test drug permeability. “It's incredibly important work,” says Arizona's Davis.

Scientists have a long way to go to understand the basic biology of many brain diseases, and a great deal of basic neuroscience research is still needed, Jacobs says. But experimental methods of crossing the blood-brain barrier form a crucial part of translating the research to the clinic. As such research picks up steam, the stakes are high. “It's high risk, high gain,” says NIH's Jacobs. “If we are successful, many, many people will benefit.”
